# Assessment during Covid-19: quality assurance of an online open book formative examination for undergraduate medical students

**DOI:** 10.1186/s12909-022-03849-y

**Published:** 2022-11-15

**Authors:** Javeria Rehman, Rahila Ali, Azam Afzal, Sara Shakil, Amber Shamim Sultan, Romana Idrees, Syeda Sadia Fatima

**Affiliations:** 1 Department for Educational Development and Department of Pathology & Laboratory Medicine, The Aga Khan University, Stadium Road, P.O. Box 3500, Karachi, 74800 Pakistan; 2 Department for Educational Development, The Aga Khan University, Karachi, Pakistan; 3 Department for Educational Development and Department of Medicine, The Aga Khan University, Karachi, Pakistan; 4 Department for Educational Development and Department of Surgery, The Aga Khan University, Karachi, Pakistan; 5 Department of Pathology & Laboratory Medicine, The Aga Khan University, Karachi, Pakistan; 6 Department of Biological and Biomedical Sciences, The Aga Khan University, Karachi, Pakistan

**Keywords:** Open book, Assessment, Quality, Medical education, COVID-19

## Abstract

**Background:**

The spread of COVID-19 pandemic in early 2020 compelled all the educational activities, including medical education to be shifted from face-to-face interaction to a virtual platform. This shift provided opportunities for exploring online assessment modalities. One such assessment method is an online open book exam which is a unique concept in medical education of Pakistan. Limited information is available in literature regarding open book exam for the basic science subjects. Hence, the objective of the study was to determine the quality of the open book exam administered as a pilot project to the first-year medical students.

**Methods:**

It was a cross-sectional analytical study that included 99 students of first year MBBS. The students were administered an online unrestricted type of open book exam as a formative assessment. The exam consisted of 30 open-ended, short answer type questions. The scores of the exam were analyzed for psychometric quality.

**Results:**

The mean score was 47.24 ± 15.30 SD %. The reliability of the exam was 0.79. The majority (66.6%) of items were found to be moderately difficult with their difficulty index ranging from 31 to 80%. The majority (86.6%) items were in the range of moderate to high discrimination. There were no questions with negative discrimination.

**Conclusions:**

The exam was found to be reliable and can be implemented with training of faculty and students. Online open book exam provides a good format for remote and formative assessment of students with minimum proctoring during times of constraints such as COVID-19 pandemic.

**Supplementary Information:**

The online version contains supplementary material available at 10.1186/s12909-022-03849-y.

## Background

The coronavirus disease (COVID-19) pandemic has impacted lives of people globally in 2020 and continues to do so [1]. With the spread of pandemic, the world succumbed to quarantine and lockdowns affecting the economic conditions, education and healthcare systems [1, 2]. Academic institutes were closed and various other social distancing practices were adopted to prevent the spread of this highly transmissible virus [1, 2]. The closure of educational institutes posed a great challenge for educators and students in terms of teaching, learning and assessment [1, 3]. The undergraduate and postgraduate medical education shifted from face-to-face interaction to a virtual platform [1, 3]. While health professional educators and learners have rapidly adapted to online learning environment, assessment particularly high-stake online examination remains a matter of concern [3, 4]. Test security and academic malpractice such as use of unfair means, plagiarism and cheating were identified as a major concern predominantly for high stake online exams [3–5].

With the paradigm shift from face to face to online, technology provided the opportunity of exploring various methods of online assessment [3]. One such method of assessment is an open-book exam (OBE) or open resource exam [3, 5]. An OBE is a type of assessment method in which the students are allowed to consult approved resources while attempting the examination [6, 7]. It may be of restricted or unrestricted format based on permission to utilize limited or unlimited resources, respectively; the resources may vary from printed material, textbooks, and personal notes to access to web and internet etc [6, 7].

Literature supports the use of OBE in clinical science subjects for undergraduate medical students [2, 4, 8]. However, with limited data from studies in basic sciences and lack of definitive psychometric evidence, its preferred use over traditional closed book format remains inconclusive. In Pakistan, to the best of our knowledge; there have been no studies that have reported the implementation and quality of OBE test formats in health professions education.

The design of OBE is essentially aimed towards testing the application of knowledge and higher order thinking skills of the students [6, 7]. The format discourages “rote-memorization” and simple transfer of information from book to the answer sheet but encourages critical thinking [6, 7, 9]. Hence, items in an efficiently designed OBE cannot be answered by simply consulting the allowed resources whether limited or unlimited [6, 7]. OBE has an advantage that it may or may not be proctored, can be taken physically in-class or virtually online or can even be administered as take-home assignments [6–8, 10]. Although OBE may be longer than the traditional closed book format, the exam duration must be sufficiently time-bound [9, 11]. OBE is perceived to reduce exam anxiety and promote deep learning [7, 9].

An online OBE can promote learners’ ability to search and translate, identify and apply evidence-based information effectively [4]. Moreover, introducing OBE to undergraduate and postgraduate medical students can motivate them as self-directed learners in times of exponentially growing pool of knowledge [4]. While the traditional closed-book format remained the preferred and predominant method of assessment globally, an online OBE emerged as a remote assessment tool in medical education during this pandemic [3, 4]. However, studies recommend to introduce OBE as a low-stake assessment to the students to familiarize them with the concept and reduce apprehensions [4].

The undergraduate curriculum at the Aga Khan University for the 1st and 2nd year MBBS is founded on problem-based learning (PBL). The curriculum is structured around a series of modules, each integrating the disciplines of anatomy, physiology, biochemistry, pharmacology, pathology, and community health sciences. To familiarize the faculty and students with the format and determine the quality of an online OBE, we embraced the opportunity to pilot a formative remote OBE for first year medical students at a private medical institution in Karachi, Pakistan. The objective of this study was to determine the quality (in terms of its reliability, difficulty index, discrimination index) of an online open-book formative exam administered as a pilot project to the first year medical students.

## Methods

### Study design and sampling

Our study was a cross-sectional analytical study design, and a total comprehensive sampling strategy was utilized for this study. Participants comprised of 99 students of first year Bachelor of Medicine, and Bachelor of Surgery (MBBS) studying in Aga Khan Medical College, Karachi. All the students of first year MBBS who appeared in this formative assessment were included.

### Exam development

The students were administered a mid-modular formative assessment in Renal module. Items were previously part of the question bank but were removed from the bank and modified for this online formative exam. The exam was developed according to a table of specifications but did not have any weightage in their summative assessment. The exam items consisted of scenario-based integrated short answer questions that focused on assessing knowledge application. An additional file shows a few items that were used in online formative OBE [see Additional file 1]. A pre-exam review of the paper was done by a team of content experts and medical educationists and all items were reviewed for quality assurance before the administration of assessment.

### Exam implementation & data confidentiality

All the students were informed about the type, schedule and format of this exam including the allowed resources. The un-proctored assessment was conducted online using Learning Management System (LMS) on open-book format and included 30 scenario-based short answer questions for a timed window of 70 minutes. The order of items was randomized for individual students’ tests. The students’ scores of the assessment were collected electronically for analysis and feedback.

Exam was scored by a single expert, trained rater according to pre-determined rubrics which underwent two tiered (content and multidisciplinary) reviews. Each item was given a score according to the desired response as mentioned in the exam key. Assessment data was secured in the exam cell and was made accessible to the investigators on request.

The exam data was shared anonymously with the researchers without any identification of the students, maintaining confidentiality of the participants. Thus, anonymity and confidentiality of the participating students and their data was ensured.

### Data analysis and post exam dissemination

Post exam analysis was done for the formative exam items. Item scores were analyzed on Statistical Package for Social Sciences version 21 to determine the mean score with standard deviation (SD), with psychometric analysis including reliability, difficulty, discrimination index of the assessment. Item and overall reliability were calculated using Cronbach’s alpha(α).

The results of post item analysis were shared in a meeting with medical educationists, module chair, coordinator, and year chair. After review of the results of post item analysis, a consensus was reached among the members and three low performing items were removed from the final results as they were found to have minor technical flaws that would affect students’ interpretation of the item. After analysis, feedback was given to students based on the item scores to improve their learning. Item performance was also valuable feedback to the module developers.

## Results

A total of 99 students appeared in the exam. The students’ scores for 30 items were analyzed. The items included were from disciplines of anatomy, physiology, biochemistry, pathology, pharmacology and community health sciences. The mean percent score was 47.24 ± 15.30 SD %. Overall item reliability of exam was moderate (α = 0.79).

**Fig. 1:**
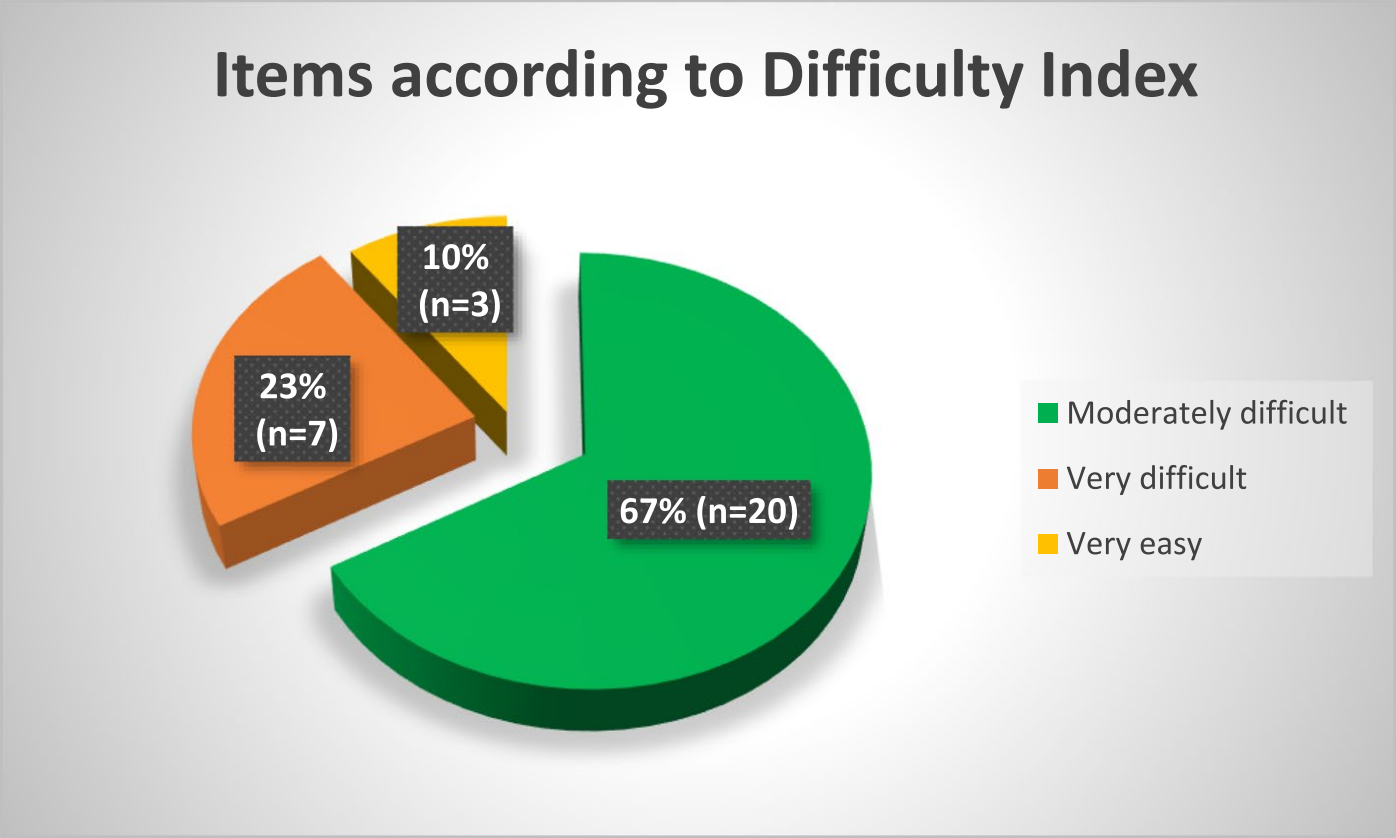
Number of Items according to Difficulty Index

Overall, the test was moderately difficult as majority of the items were found to be of moderate difficulty with difficulty index between 31 and 80% as shown in Fig. 1: Number of Items according to Difficulty Index. While 23.3% of the items were identified as very difficult with their difficulty indices of less than 30%, few were very easy with their difficulty indices of more than 80%. An additional file shows the item analysis in more detail [see Additional file 2].

**Fig. 2:**
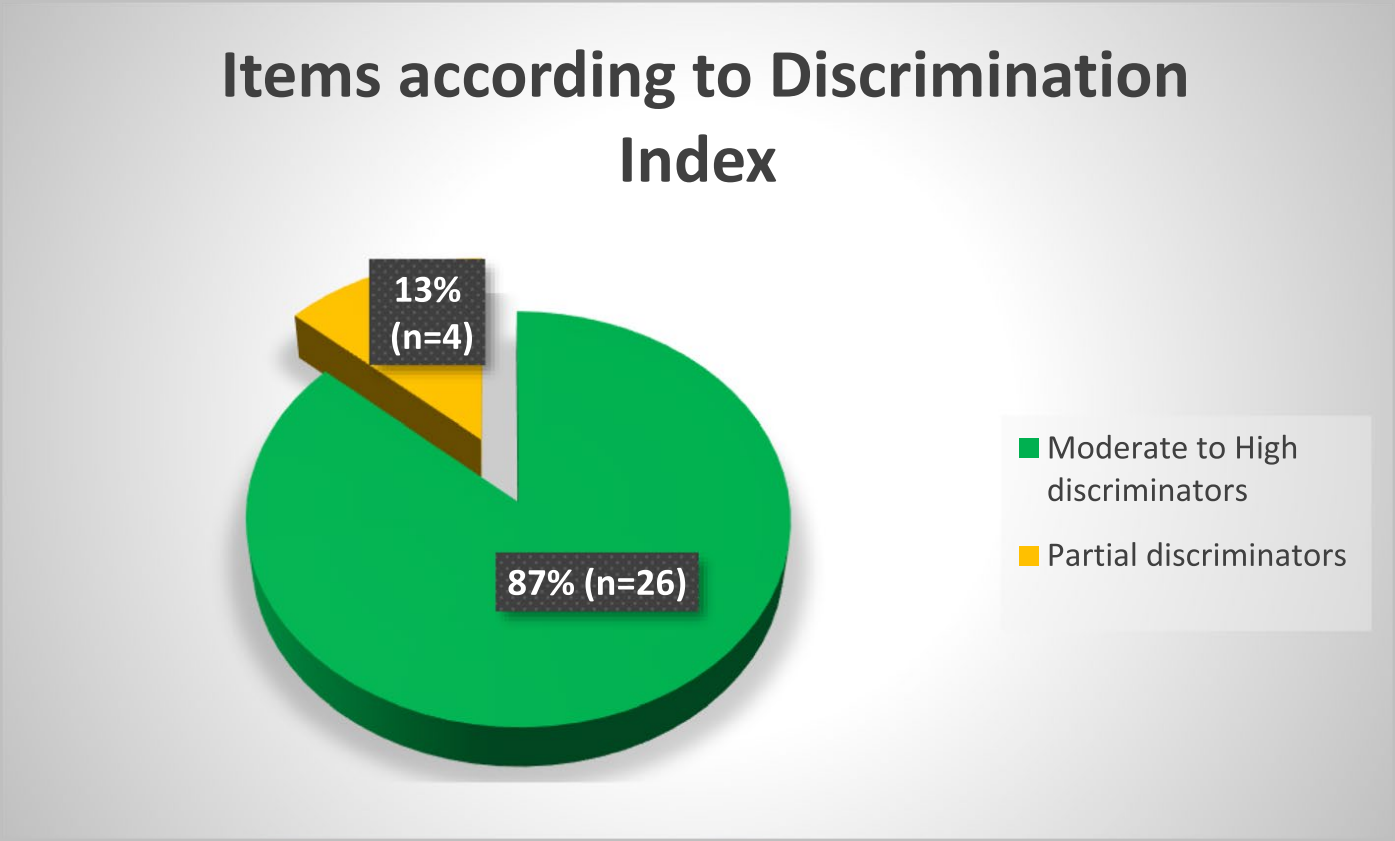
Number of Items according to Discrimination Index

The OBE was highly discriminatory between high performing and low performing students as majority of the items were found to have moderate (index ranging from 16 to 30) to high discrimination indices of more than 30. There were no items with negative discrimination as shown in Fig. 2: Number of Items according to Discrimination Index.

## Discussion

With the onset of COVID 19, along with the teaching and learning strategies the assessment strategies had to be redesigned to ensure a fair assessment system with minimal risk to all concerned stakeholders. At the Aga Khan University, measures were taken to move the assessment system to an online platform with proctoring to ensure fairness. Another option of online OBE was considered; an analysis was undertaken to determine applicability, feasibility and quality of the assessment for an online formative exam of renal module before its implementation.

The results show that OBE is a reliable method of assessing students to understand, interpret and apply the taught concepts. Psychometric quality of a well-constructed open book assessment can be reached alongside the traditional closed book exams without much difference [12]. The recommended range of reliability in a formative exam is 0.7–0.79, 0.8–0.89 in summative for moderate stake and 0.9 and above for very high stake licensure or certification exam in medical education [13]. The reliability of OBE in our study was determined to be moderate with α = 0.79 which is in accordance with the recommended range. This value can be considered proximate not only to the recommended range of summative exam but also to the reliability of α = > 0.8 as reported by Sam et al. in a high stake summative open book exam for final year [14].

OBEs allow educators to move from assessment of rote memorization to that of higher-order cognitive skills and critical thinking [9, 15, 16]. All items of the OBE administered to the students in our study were of open ended, short answer format, essentially assessed higher order thinking with application of knowledge and were targeted at reasoning skills including analysis and interpretation. Short answer questions (constructed response) assessing application of knowledge minimize the chance for guessing, are less searchable, help to uncover learners’ reasoning and challenge their thinking [17–19]. The high reliability of OBE in this study suggests that it is possible to have satisfactory reliability with short answer questions during open book exams. Hence, equal or higher test reliabilities can be achieved with fewer short answer questions advocating that short answer tests are efficient if comparable reliabilities are to be attained [20, 21]. The use of open-ended type questions in our study is in accordance with a number of studies such as by Moore [22], Vidya [23] and Erlich [24] and Krasne et al [25] However, various studies have also reported the use of selected response items such as multiple choice, one-best answer type questions or a combination of both selected and constructed response type items, in open book exams [2, 26–29]. Item analysis in this study showed that distribution of items, in terms of their level of difficulty, was fairly balanced with majority of the questions being moderately difficult. Comparable difficulty and discrimination indices of OBE with closed book format are reported by others [14, 25]. Moreover, clearly written unambiguous test questions of medium difficulty, which improve reliability of assessment, are again supportive of our findings [13]. The OBE items in this study were highly discriminatory between high performing and low performing students as 86.6% of the items were found to have moderate to high discrimination indices. This finding is in similarity with other studies which observed that tests with open-ended items can better discriminate between well-prepared students and marginal students, as they allow more possibility for differentiation in scores [21, 30, 31].

The low mean percent score of the students in this study could be possibly due to the lack of experience in-terms of preparation, effective utilization and attempt of OBE by the students. However, studies have reported low scores, high scores, or no difference in scores of students in OBE when compared to the closed book exam (CBE) in literature [9, 12, 25, 32].

### Implications

Being mid-module formative assessment, the online OBE helped students to be provided with timely feedback and seek needed support in times of constraints such as during COVID-19. This format provided an opportunity for learners’ self-analysis to identify areas for improvement as well as an opportunity for faculty to reflect and reinforce the essential concepts where the learner might be struggling.

The online administration of exam through LMS was also found to be practically feasible, cost-effective, time efficient and required minimum proctoring.

### Limitations and future research direction

One of our study limitations was that both the faculty and students had no prior training or experience in OBE. For faculty, this limitation was taken care of with support of medical educationists in offering them guidance and reviewing the questions for attaining the desired quality of assessment. Students were also briefed about the format of the exam.

Future research is needed to compare the findings for summative open book exam and to further evaluate its impact on desired students’ outcome.

## Conclusion

The quality of online open-book formative exam administered to the first-year medical students was assured. The exam was found to be reliable and can be implemented with training of faculty and students. Online OBE provided a feasible format for remote and formative assessment of students with minimum proctoring during times of constraints such as COVID-19 pandemic.

The findings of the study suggest that a well-constructed, good quality formative OBE can be implemented online to provide timely and effective feedback for students’ learning. Moreover, this online OBE format can be used for future assessments including summative exams with appropriate standard setting methods. Faculty training to familiarize module faculty members with the online OBE format will enhance their capacity to apply it in their respective modules. Furthermore, students should be trained to utilize resources effectively while preparing for and attempting OBE.

## Supplementary Information

**Additional file 1.** Shows few items that were used in online formative OBE.

**Additional file 2.** Shows table of item analysis results of online formative OBE.

## Data Availability

Datasets analysed during this study are included in this published article and its supplementary information files.

## Abbreviations

OBE: open book exam
COVID-19: coronavirus disease of 2019
MBBS: Bachelor of Medicine, and Bachelor of Surgery
PBL: problem-based learning
SD: standard deviation
CBE: closed book exam
LMS: Learning management system
