# Supplementary material for: Assessment during Covid-19: quality assurance of an online open book formative examination for undergraduate medical students
Source: BMC Med Educ. 2022 Nov 15;22:792. doi: 10.1186/s12909-022-03849-y (PMC9666962; doi:10.1186/s12909-022-03849-y)
Supplement: Supplementary file 1 — Additional file 1. Shows few items that were used in online formative OBE. [file 12909_2022_3849_MOESM1_ESM.pdf]

Examples of Short Answer Questions (SAQs) used in OBE

Scenario: After playing in a local garbage dump, a 5-year-old boy develops a small abscess on his arm. 3 weeks later he presents to a local GP with complaints of malaise, fever, nausea, and smoky brown urine. Examination and investigation revealed oliguria, oedema, azotaemia and increased blood pressure. Urine detailed report showed proteinuria. Blood uric acid was sent for lab analysis.

Q1. What is the underlying mechanism of increased blood pressure in this case? (2 marks)

Q2. What is the effect of the above-mentioned renal symptoms on uric acid levels? Justify your answer. (2 marks)

Q3. Which transport mechanism is disrupted in this case? Relate with the patient`s findings. (2 marks)

Q4. In this patient, the injury in the involved organ is found at which microscopic site? (1 mark)

Scenario (cont.): As the patient presented with oedema and hypertension due to sodium retention leading to fluid overload, the physician decided to give him a drug that would increase the excretion of sodium and fluid by increasing the volume of urine.

Q5. Which drug will be most suitable in this case keeping in view that a strong and immediate action is required? (1 mark)

Q6. Give the rationale of your choice of drug in this case by relating to its mode of action. (3 marks)

Scenario (cont.): Percutaneous renal biopsy was performed to establish a diagnosis for this patient.

Q7. What would be the most pertinent morphological findings on light microscopy? (3 marks)
