# Supplementary material for: Assessment during Covid-19: quality assurance of an online open book formative examination for undergraduate medical students
Source: BMC Med Educ. 2022 Nov 15;22:792. doi: 10.1186/s12909-022-03849-y (PMC9666962; doi:10.1186/s12909-022-03849-y)
Supplement: Supplementary file 2 — Additional file 2. Shows table of item analysis results of online formative OBE. [file 12909_2022_3849_MOESM2_ESM.pdf]

ITEM ANALYSIS RESULT

FORMATIVE ONLINE OPEN BOOK EXAM

Before Analysis	
Mean%	47.24
SD%	15.30
Reliability	0.791

(del Q.21,28,29)	
Mean%	49.28
SD%	16.07
Reliability	0.804

Number of questions in the examination: **MCQs: 30**

	DISCRIMINATION						
**DIFFICULTY**	**# Qs**	**(<-15)**	**(-15 to -0.5)**	**0**	**(+0.5 to +15)**	**(+16 to +29)**	**(>=30)**
**0-30%**	**7**	0	0	1	2	2	2
				28,	29,21,	23,27,	30,10,
**31-80%**	**20**	0	0	0	1	4	15
					19,	1,4,26,5,	12,15,13,17,25,14,11,22,3,20,16,2,9,7,6,
**81-100%**	**3**	0	0	0	0	2	1
						8,24,	18,
**Total**	**30**	**0**	**0**	**1**	**3**	**8**	**18**

Discrimination Index:
High Negative: (< -15), Negative: (-15 to - 0.5), None: (0), Positive: (+0.5 to +15) Moderate (+16 to +29), High: (>30)

Difficulty index:
very difficult (0-30%), moderately difficult (30-80%), very easy (>80%)
